# Family Leave and Return-to-Work Experiences of Physician Mothers

**DOI:** 10.1001/jamanetworkopen.2019.13054

**Published:** 2019-10-11

**Authors:** Shannon B. Juengst, Alexa Royston, Isabel Huang, Brittany Wright

**Affiliations:** 1 Department of Physical Medicine and Rehabilitation, University of Texas Southwestern Medical Center, Dallas

## Abstract

**Importance:**

With the substantial increase in the proportion of women graduating from medical school, factors surrounding family leave require careful attention. Although many circumstances and experiences are unique to the training setting and specialty, a nationwide representation of physician mothers across all disciplines and all levels of training may reveal common themes and experiences among them, enable comparison across training setting and subspecialties, and identify best practices for supporting physician mothers throughout their careers.

**Objective:**

To characterize family leave and return-to-work experiences of physician mothers across subspecialties.

**Design, Setting, and Participants:**

This cross-sectional US nationwide survey study evaluating the experiences of 844 physician mothers was administered electronically via REDCap from September 2 to December 20, 2018.

**Main Outcomes and Measures:**

A survey was developed using a modified Delphi process with a panel of experts to characterize physician mothers’ family leave and return-to-work experiences. The survey covered both negative and positive experiences of physician mothers for each child they conceived or adopted after medical school to identify areas for change and strategies for successfully supporting physician parents.

**Results:**

Of 1465 potentially eligible survey respondents, 844 (57.6%) were verified as unique respondents with complete surveys. Their mean (SD) age was 35.8 (5.2) years (range, 27-67 years), with most women (826 [97.9%]) currently practicing and 138 women (16.4%) currently in a residency program. Participants were included from 19 subspecialty groups. Of the women surveyed, 619 (73.3%) felt that leave time was insufficient. The majority (751 [89.0%]) would have preferred 11 weeks to 6 months of leave vs the 5 to 12 weeks (often not paid) most commonly available. The most frequently reported negative experiences when returning to work were associated with lack of facilities for breast pumping (range, 12 of 78 [15.4%] for the third child to 272 of 844 [32.2%] for the first child) and time for breast pumping (range, 27 of 78 [34.6%] for the third child to 407 of 844 [48.2%] for the first child), difficulty obtaining childcare (eg, for the first child, 298 of 844 [35.3%]), and discrimination (eg, for the first child, 152 of 844 [18.0%]). The most common positive experience was emotional support (eg, for the first child, 504 of 844 [59.7%]), primarily from colleagues.

**Conclusions and Relevance:**

The present study, capturing one of the largest and most heterogeneous samples of physician mothers, showed that a substantial number of women physicians working in a variety of specialties at all levels of training across the United States needed and wanted more support for maternity leave and return to work. Support at the institutional level, such as paid leave, adequate breast pumping time without penalty, on-site childcare, and schedule flexibility, would likely provide the greatest direct assistance to help physician mothers thrive in their careers.

## Key Points

**Question:**

What do physician mothers experience when taking maternity leave and returning to work?

**Findings:**

In this cross-sectional survey study, 844 physician mothers across multiple subspecialties reported positive and negative experiences associated with taking leave, returning to work, breastfeeding or breast milk pumping, childcare, perceived discrimination, and important supportive factors.

**Meaning:**

This study’s findings suggest that physicians taking maternity leave experience unique challenges that require creative solutions, the seeds of which can be found in the lived experiences of women in medicine captured in the present study.

## Teaser

This cross-sectional US nationwide survey study assesses the negative and positive aspects of family leave and return-to-work experiences among physician mothers to identify best practices for supporting physician parents.

## Introduction

Nearly all physicians find achieving work-life integration difficult because of long work hours and uncompromising schedules.^1,2^ With substantial growth in the proportion of women graduating from medical school,^3^ factors surrounding maternity/family leave require particular attention. Current family medical leave policies in the United States are inadequate and often difficult to put into effective practice in medicine.^4^ Physician mothers frequently stitch together a patchwork of sick days, vacation, and unpaid time off to recover, provide care, and bond with their child before returning to work, often leading to career dissatisfaction and burnout.^1,2,5,6^

In the United States, attending physicians and residents actually take a mean of 8 weeks and 6 weeks, respectively, of paid maternity leave.^7,8,9,10,11,12^ Leave time policies are inconsistent and often come with several caveats and constraints under the discretion of institutional and departmental leadership. Physician mothers who take maternity leave receive lower peer evaluation scores, lose thousands of dollars of potential income, are penalized with increased call both before and after leave, and often encounter maternal discrimination in the workplace.^4,9,13,14,15,16^

Several studies have characterized maternity leave trends among physician mothers using surveys distributed via social media. Many have focused on a single training setting (eg, residency vs attending) or specialty (eg, surgical vs nonsurgical) and rarely used rigorous survey development methods.^6,8,13,14,17^ A nationwide survey of physician mothers across levels of training and multiple subspecialties could reveal common themes and experiences among physician mothers, enable comparisons across training settings and subspecialties, and identify best practices and strategies for supporting physician mothers throughout their careers. To this end, we used rigorous survey development methods to create and administer a survey that characterized maternity/family leave and return-to-work experiences of physician mothers across the United States.

## Methods

We conducted a cross-sectional nationwide survey study of physician mothers’ experiences with family/maternity leave and return to work for each child born or adopted after completion of medical school. We developed the detailed survey using a modified Delphi process with input from a diverse panel of experts composed of physicians across multiple specialties, a nurse, a lawyer, and a policy expert. The survey characterizes workplace settings and experiences during pregnancy, maternity leave, and return to work for mothers of all children born or adopted after completion of medical school, representing the most comprehensive survey on this topic to our knowledge. All procedures were approved by the University of Texas Southwestern Medical Center, Dallas, Institutional Review Board, which granted a waiver of written informed participant consent because this was an anonymous survey study. However, all participants indicated consent to participate in the study. This study followed the Strengthening the Reporting of Observational Studies in Epidemiology (STROBE) reporting guideline.

The survey contained 12 questions characterizing participant demographics and clinical practice settings (these were repeated for each child only if they differed from the initial responses). Per child, the survey also included 102 questions about pregnancy or adoption planning and history, family composition, and birth or adoption experiences; 61 questions about maternity or paternity leave experiences; and 68 questions about return-to-work experiences. Response options included yes or no or multiple choice options, with free text available when participants selected the option of “other.”

### Participants and Recruitment

The anonymous survey was administered electronically via REDCap (a Health Insurance Portability and Accountability Act–compliant web-based data collection platform) from September 2 to December 20, 2018, via the American Medical Women’s Association email listserv, multiple Facebook groups specifically for physician mothers (eg, Physician Mom Group, Dr MILK), Twitter, LinkedIn, and personal social media pages. Inclusion criteria were identifying as being a mother, a physician (resident and nonresident physicians both included), and fluent in English.

### Statistical Analysis

We present descriptive statistics (frequencies and percentages) characterizing our sample and participants’ experiences as women in medicine with maternity/family leave and return to work. All data were analyzed using SPSS software, version 24 (IBM Statistics).

## Results

### Recruitment

**Figure 1.**
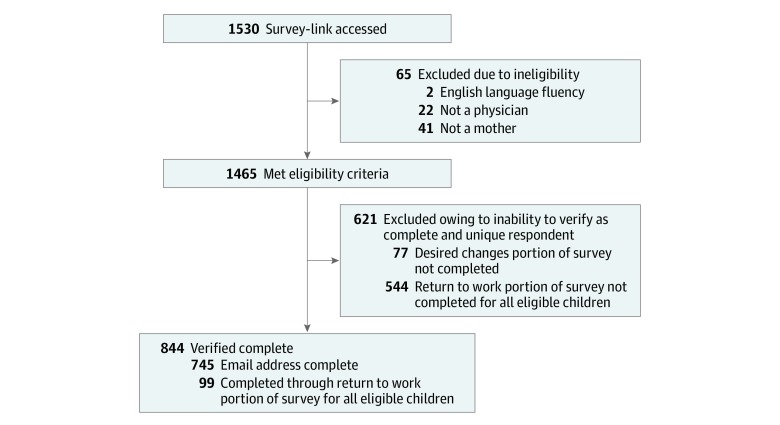
Flowchart of Included and Excluded Surveys and Reasons for Exclusion in the Final Data Set

Figure 1 is a flowchart of surveys included and excluded from the present study, describing reasons for ineligibility, incomplete surveys, and final survey completion numbers. For data quality purposes, we took a conservative approach and included only surveys we could verify as being complete and belonging to unique respondents. Of the 1465 surveys initiated by eligible participants, we verified that 844 (57.6%) were unique respondents with complete surveys.

### Characterization of Participants

**Figure 2.**
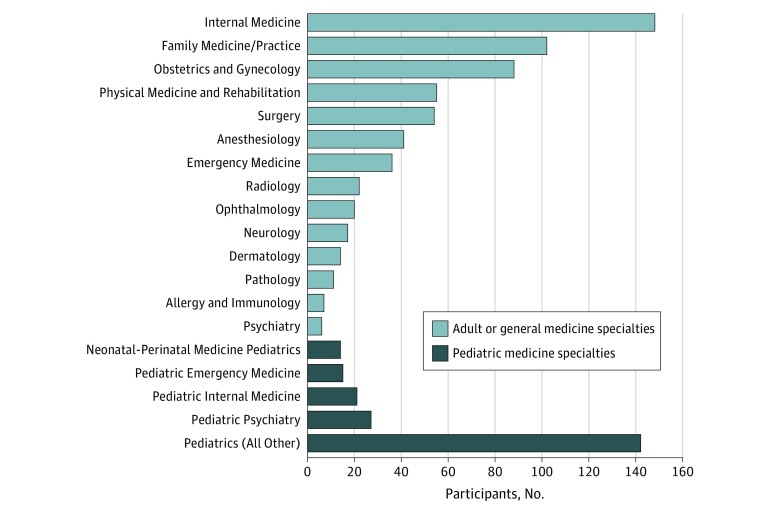
Specialties of Survey Participants Number of participants in each of 19 specialty categories from which participants could choose. Categories were created from a more detailed list of 130 specialties.^18^ The “Pediatrics (All Other)” category includes 90 “pediatrics” and any other pediatric specialty represented by 7 or fewer participants.

Table 1 describes participants’ demographics, current training and practice settings, years practicing, and numbers of pregnancies and children. The mean (SD) age of the 844 respondents was 35.8 (5.2) years (range, 27-67 years). Figure 2 presents the frequency of respondents in 19 subspecialties.^18^ Notably, almost all participants (756 [89.6%]) were within their first 10 years of practice, indicating that these results largely represent recent experiences of physician mothers. Most (826 [97.9%]) were currently attending physicians, with only 138 currently in residency training (16.4%). Nearly one-third (247 [29.3%]) experienced a miscarriage, and 10 women (1.2%) adopted at least 1 child. A substantial majority (768 [91.1%]) had only 1 or 2 children, with most women (743 [88.0%]) having their first child after completing medical school.

**Table 1.** Characteristics of Physician Mothers

Characteristic	No. (%) of Respondents
No.	844
Age, mean (SD) [range], y	35.8 (5.2) [27-67]
Currently	
Practicing	826 (97.9)
Resident	138 (16.4)
International medical graduate	79 (9.4)
Years in practice	
0-5	530 (62.8)
6-10	226 (26.8)
11-15	49 (5.8)
16-20	22 (2.6)
>20	17 (2.0)
Current work status	
Employed full time	694 (82.2)
Employed part time	103 (12.2)
Unemployed	15 (1.7)
On medical/family leave	27 (3.2)
On disability or retired	5 (0.6)
Mean time worked/wk, h	
<25	31 (3.7)
25-40	214 (25.4)
41-60	446 (52.8)
61-80	129 (15.3)
>80	24 (2.8)
Current or most recent practice setting	
Solo practice	11 (1.3)
Private practice group ≤3 physicians	16 (1.9)
Private practice group >3 physicians	143 (16.9)
Hospital-based practice	235 (27.8)
Academic setting	390 (46.2)
Other	49 (5.8)
Ever carried a pregnancy to successful delivery	832 (99.3)
Experienced a miscarriage	247 (29.3)
Able to take time off to recover from miscarriage	97 (39.3)
Would have benefited from being able to take time off	114 (76.0)
Ever adopted a child/children	10 (1.2)
International	4 (0.5)
Domestic	6 (0.7)
Needed additional time off for adoption process	7 (70.0)
Total No. of children	
1	449 (53.3)
2	319 (37.8)
3	69 (8.2)
4	6 (0.7)
First child conceived or adopted after completing medical school	
First	743 (88.0)
Second	41 (4.9)
Third	6 (0.7)
Fourth	2 (0.2)
Unknown (question introduced after survey completion)	52 (6.2)
Had someone recommend she not become pregnant at a particular period of time in training or work	404 (47.9)
Delayed plans for family as a result	128 (15.2)
Regretted delaying family planning	47 (5.6)
Felt delay contributed to future difficulties conceiving	48 (5.7)
Felt encouraged to have children/become pregnant at time course of her choosing	454 (53.8)

### Family Leave and Return-to-Work Experiences of Physician Mothers by Child

Table 2 provides a detailed characterization of maternity and partners’ paternity leave experiences, broken down by first, second, and third child. Across all children, only about half of participants (range, 390 of 844 [46.2%] for the first child to 41 of 79 [51.9%] for the third child) reported having paid maternity/family leave offered through their employer benefits. The primary source for maternity leave was sick time (range, 36 of 79 [45.6%] for the third child to 202 of 389 [51.9%] for the second child), followed by accrued paid time off (range, 34 of 79 [43.0%] for the third child to 190 of 389 [48.8%] for the second child). Only approximately one-quarter to one-third of respondents (range, 225 of 844 [26.7%] for the first child to 30 of 79 [38.0%] for the third child) felt that the amount of leave time available was sufficient, with the vast majority of respondents indicating they would have preferred either 11 to 12 weeks (269 of 844 [31.9%] for the first child; 124 of 389 [31.9%] for the second child; and 27 of 79 [34.2%] for the third child) or 4 to 6 months (360 of 844 [42.7%] for the first child; 185 of 389 [47.6%] for the second child; and 32 of 79 [40.5%] for the third child). Approximately one-third of respondents (327 of 844 [38.7%] for the first child; 119 of 389 [30.6%] for the second child; and 26 of 79 [32.9%] for the third child) were less than satisfied with their maternity leave experience, and more than two-thirds of respondents with a partner (523 of 802 [65.2%] for the first child; 230 of 358 [64.2%] for the second child; and 41 of 71 [57.7%] for the third child) were dissatisfied with the paternity leave available to their partner. Financial contraints were the most frequently cited factor affecting duration of leave (308 of 844 [36.5%] for the first child; 177 of 389 [45.5%] for the second child; and 37 of 79 [46.8%] for the third child), followed by need to return to clinical responsibilities (287 of 844 [34.0%] for the first child; 142 of 389 [36.5%] for the second child; and 28 of 79 [35.4%] for the third child), and personal preference to maximize leave time (214 of 844 [25.4%] for the first child; 107 of 389 [27.5%] for the second child; and 20 of 79 [25.3%] for the third child). For the first children, threat of prolonged training was among the most frequently cited reasons affecting leave duration (273 of 844 [32.3%]). Furthermore, many women reported that they had to make up time (eg, extra calls, extra clinic time) taken for leave (308 of 844 [36.5%] for the first child; 108 of 389 [27.8%] for the second child; 16 of 79 [20.3%] for the third child). Although less common, many women also experienced verbal pressure to return to work earlier (55 of 844 [6.5%] for the first child; 34 of 389 [8.7%] for the second child; 10 of 79 [12.7%] for the third child) and verbal admonishment for the increased workload on covering physicians (114 of 844 [13.5%] for the first child; 52 of 389 [13.4%] for the second child; 10 of 79 [12.7%] for the third child). The most common positive experiences, reported by more than half of the respondents, were visits from coworkers (481 of 844 [57.0%] for the first child; 213 of 389 [54.8%] for the second child; 40 of 79 [50.6%] for the third child) and uninterrupted time to focus on their new child (459 of 844 [54.4%] for the first child; 218 of 389 [56.0%] for the second child; 38 of 79 [48.1%] for the third child). Most respondents (775 for child 1, 357 for child 2, 72 for child 3) reported that paternity/domestic partner leave was either not available or not otherwise taken by their spouse/coparent/domestic partner. Among first-time mothers, 259 of 844 (30.7%) did not feel that they had adequate information about family leave options.

**Table 2.** Physician Mother Experiences by Child

Experience	No. (%) of Respondents		
	Child 1	Child 2	Child 3
**During Pregnancy**^**a**^			
No.	844	394	81
Pregnancy with no intervention	727 (86.1)	349 (88.6)	69 (85.2)
Intervention-assisted pregnancy	111 (13.2)	42 (10.7)	11 (13.6)
Adoption	6 (0.7)	3 (0.8)	1 (1.2)
Pregnancy resulting in twins/multiples	12 (1.4)	12 (3.0)	5 (6.2)
Did other physicians in group cover for workload? (yes)	688 (81.5)	346 (87.8)	78 (96.3)
Did workplace provide health insurance benefits? (yes)	791 (93.7)	366 (92.9)	73 (90.1)
**Family/Maternity Leave**			
No.	844	389	79
Year leave was taken			
1981-2000	20 (2.4)	9 (2.3)	3 (3.8)
2001-2010	111 (13.2)	50 (12.9)	8 (10.1)
2011-2020	713 (84.5)	330 (84.8)	68 (86.1)
Employer offered paid maternity/family leave	390 (46.2)	191 (49.1)	41 (51.9)
Maternity/family leave options discussed prior to leave	436 (51.7)	240 (61.7)	52 (65.8)
Aware of FMLA	641 (75.9)	326 (83.8)	65 (82.3)
Aware of deadline for FMLA submission	346 (41.0)	212 (54.5)	49 (62.0)
Given accurate information about maternity/family leave options	519 (61.5)	261 (67.1)	56 (70.9)
Had adequate information about maternity/family leave plan	585 (69.3)	301 (77.4)	57 (72.2)
Given inaccurate information on maternity/family leave	175 (20.7)	66 (17.0)	11 (13.9)
Believed this misinformation was purposefully given to minimize length of maternity/family leave taken	41 (23.4)	16 (24.2)	3 (27.3)
How long a leave was officially available			
0-2 wk	51 (6.1)	18 (4.7)	5 (6.4)
3-4 wk	42 (5.0)	7 (1.8)	0
5-6 wk	121 (14.3)	52 (13.4)	5 (6.3)
7-10 wk	112 (13.2)	32 (8.2)	4 (5.1)
11-12 wk	328 (38.9)	178 (45.8)	48 (60.8)
4-6 mo	44 (5.2)	23 (5.9)	4 (5.1)
>6 mo	7 (0.9)	4 (1.1)	0
Unknown/not sure	139 (16.5)	75 (19.3)	13 (16.5)
Took paid maternity/family leave			
Yes, for full leave	205 (24.3)	92 (23.7)	13 (16.7)
Yes, for part of leave	240 (28.4)	129 (33.2)	30 (38.5)
No	399 (47.3)	168 (43.2)	35 (44.9)
Source of maternity leave days			
Sick leave	406 (48.1)	202 (51.9)	36 (46.2)
Holiday carryover	179 (21.2)	68 (17.5)	13 (16.7)
Paid time off	388 (46.0)	190 (48.8)	34 (43.6)
Specified maternity leave	261 (30.9)	134 (34.4)	31 (39.7)
Pooled general leave days	93 (11.0)	38 (9.8)	6 (7.7)
Other	282 (33.4)	116 (29.8)	28 (35.9)
How much time off was paid?			
0-2 wk	30 (3.5)	21 (5.4)	5 (6.4)
3-4 wk	58 (6.9)	22 (5.7)	4 (5.1)
5-6 wk	139 (16.5)	60 (15.4)	21 (26.9)
7-10 wk	123 (14.6)	62 (15.9)	6 (7.7)
11-12 wk	76 (9.0)	45 (11.6)	5 (6.4)
4-6 mo	9 (1.1)	8 (2.1)	2 (2.6)
>6 mo	1 (0.1)	2 (0.5)	0
Unknown	9 (1.1)	1 (0.3)	0
None	399 (47.3)	168 (43.2)	35 (44.9)
Thought leave time was sufficient	225 (26.7)	145 (37.3)	30 (38.5)
Felt there was adequate support for maternity/family leave plan	501 (59.4)	269 (69.2)	55 (70.5)
How long a maternity/family leave would you have preferred?			
0-2 wk	0	0	1 (1.3)
3-6 wk	17 (2.1)	11 (2.8)	3 (3.8)
7-10 wk	67 (8.0)	27 (7.0)	4 (5.1)
11-12 wk	296 (35.1)	124 (31.9)	27 (34.6)
4-6 mo	360 (42.7)	185 (47.6)	32 (41.0)
>6 mo	95 (11.3)	37 (9.5)	11 (14.1)
Unknown	9 (1.1)	5 (1.3)	0
Factors affecting duration of leave			
International student limited by work visa	14 (1.7)	5 (1.3)	0
Personal preference to return to work earlier	67 (7.9)	48 (12.3)	8 (10.3)
Personal preference to maximize leave time	214 (25.4)	107 (27.5)	20 (25.6)
Research grant/fellowship requirement	73 (8.6)	26 (6.7)	3 (3.8)
Threat of prolonged training	273 (32.3)	41 (10.5)	3 (3.8)
Need to return to clinical responsibilities	287 (34.0)	142 (36.5)	28 (35.9)
Pressure from employer	147 (17.4)	68 (17.5)	13 (16.7)
Pressure from colleagues	111 (13.2)	50 (12.9)	14 (17.9)
Family-related factors	33 (3.9)	20 (5.1)	3 (3.8)
Financial factors	308 (36.5)	177 (45.5)	37 (47.4)
Other	51 (6.0)	30 (7.7)	2 (2.6)
Experienced significant financial difficulties because of lack of pay during leave	155 (18.4)	89 (22.9)	22 (28.2)
What kind of support would have been most helpful			
Waiver of overhead fees	22 (2.6)	9 (2.3)	2 (2.6)
More paid leave	295 (35.0)	105 (27.0)	20 (25.6)
Paid nanny	118 (14.0)	44 (11.3)	7 (9.0)
Paid mother’s helper	81 (9.6)	31 (8.0)	3 (3.8)
Paid meal service	96 (11.4)	37 (9.5)	7 (9.0)
Paid daycare	127 (15.0)	45 (11.7)	7 (9.0)
More support from parents	96 (11.4)	28 (7.2)	1 (1.3)
More support from friends	46 (5.5)	17 (4.4)	1 (1.3)
More legal protections regarding family leave	120 (14.2)	25 (6.4)	7 (9.0)
Required to make up time taken for leave (eg, extra calls, extra clinic time)	308 (36.5)	108 (27.8)	16 (20.5)
Negative experiences			
Verbal pressure to return prior to physician recommendations	72 (8.5)	23 (5.9)	2 (2.6)
Expectation to continue administrative tasks	177 (21.0)	92 (23.7)	19 (24.4)
Expectations to answer work emails while on leave	272 (32.2)	129 (33.2)	26 (33.3)
Verbal pressure to return to work prior to negotiated time off	55 (6.5)	34 (8.7)	10 (12.8)
Termination from job	6 (0.7)	6 (1.5)	0
Verbal admonishment for increased workload on covering physicians	114 (13.5)	52 (13.4)	10 (12.8)
Discussion regarding anticipated delay in partnership status	24 (2.8)	10 (2.6)	2 (2.6)
Anger directed toward you as a result of rescheduled appointments	84 (10.0)	23 (5.9)	8 (10.3)
Derogatory words	58 (6.9)	20 (5.1)	5 (6.4)
Other	62 (7.3)	12 (3.1)	2 (2.6)
None	392 (46.4)	197 (50.6)	41 (52.6)
Positive experiences			
Visits from coworkers	481 (57.0)	213 (54.8)	40 (51.3)
Reassurance of job status	274 (32.5)	136 (35.0)	26 (33.3)
Support from staff regarding patient scheduling	216 (25.6)	108 (27.8)	25 (32.1)
Uninterrupted time to focus on self-care	281 (33.3)	140 (36.0)	27 (34.6)
Uninterrupted time to focus on new child	459 (54.4)	218 (56.0)	38 (48.7)
Other	16 (1.9)	6 (1.5)	0
None	97 (11.5)	55 (14.1)	17 (21.8)
Overall satisfaction with maternity/family leave experience			
Dissatisfied	113 (13.4)	41 (10.5)	8 (10.3)
Less satisfied	214 (25.4)	78 (20.1)	18 (23.1)
Satisfied	314 (37.2)	156 (40.1)	25 (32.1)
Very satisfied	152 (18.0)	83 (21.3)	19 (24.4)
Completely satisfied	51 (6.0)	31 (8.0)	8 (10.3)
**Paternity Leave by Child**^**b**^			
No.	844	389	78
Paternity leave/domestic partner leave offered			
Yes	321 (38.0)	123 (31.6)	21 (26.9)
No	481 (57.0)	235 (60.4)	50 (64.1)
NA (no partner involved)	42 (5.0)	31 (8.0)	7 (9.0)
Paid paternity/domestic partner leave			
Partially paid	38 (4.5)	13 (3.3)	1 (1.3)
Fully paid	230 (27.3)	89 (22.9)	15 (19.2)
Not paid at all	53 (6.3)	21 (5.4)	5 (6.4)
NA or no leave available	523 (62.0)	266 (68.4)	57 (73.1)
Given accurate information about paternity leave	397 (49.5)	197 (55.0)	40 (51.3)
Length of paternity/domestic partner leave actually taken, wk			
0	9 (1.1)	6 (1.5)	1 (1.3)
1	15 (1.8)	11 (2.8)	2 (2.6)
2	18 (2.1)	6 (1.5)	1 (1.3)
3-4	16 (1.9)	6 (1.5)	2 (2.6)
5-6	5 (0.6)	1 (0.3)	0
7-10	5 (0.6)	2 (0.5)	0
≥11	1 (0.1)	0	0
Not available or otherwise not taken	775 (91.8)	357 (91.8)	72 (92.3)
Full leave available not taken	69 (21.5)	32 (26.0)	6 (28.6)
Reasons full paternity/domestic partner leave not taken			
Threat for no time off	3 (4.3)	2 (6.3)	2 (33.3)
Inability to schedule time off when desired	17 (24.6)	9 (2.8)	1 (16.7)
Misinformation of available leave time	10 (14.5)	2 (6.3)	0
Perceived threat for termination of job	6 (8.7)	5 (15.6)	0
Verbal admonishment for increased workload on coworkers	12 (17.4)	3 (9.4)	1 (16.7)
Intrinsic feelings to not take leave because others did not take paternity/domestic partner leave	23 (33.3)	11 (34.4)	1 (16.7)
Personal preference to return to work earlier	31 (44.9)	15 (46.9)	3 (50.0)
Other	11 (1.3)	3 (9.4)	1 (16.7)
How long a paternity/domestic partner leave would you have preferred			
0 wk	30 (3.7)	21 (5.9)	4 (5.6)
1 wk	4 (0.5)	3 (0.8)	0
2 wk	69 (8.6)	43 (12.0)	12 (16.9)
3-4 wk	266 (33.2)	119 (33.2)	21 (29.6)
5-6 wk	108 (13.5)	45 (12.6)	10 (14.1)
7-10 wk	79 (9.9)	32 (8.9)	5 (7.0)
11-12 wk	123 (15.3)	48 (13.4)	6 (8.5)
4-6 mo	46 (5.7)	11 (3.1)	2 (2.8)
>6 mo	18 (2.2)	2 (0.6)	1 (1.4)
Unknown	59 (7.4)	34 (9.5)	10 (14.1)
General satisfaction with paternity leave experience			
Dissatisfied	339 (42.3)	137 (35.2)	27 (38.0)
Less satisfied	184 (22.9)	93 (23.9)	14 (19.7)
Satisfied	150 (18.7)	85 (21.9)	22 (31.0)
Very satisfied	84 (10.5)	30 (7.7)	3 (4.3)
Completely satisfied	45 (5.6)	13 (3.3)	5 (7.0)
NA	42 (5.3)	31 (8.0)	7 (9.9)

Abbreviations: FMLA, Family Medical Leave Act; NA, not applicable.

^a^ Some participants responded to questions per pregnancy rather than per child.

^b^ Percentages calculated using the total number of respondents who had leave available.

Table 3 details experiences physician mothers had when returning to work. The most frequently reported negative experiences were associated with the lack of lactation facilities (range, 12 of 78 [15.4%] for the third child to 272 of 844 [32.2%] for the first child) and time available for breast pumping (range, 27 of 78 [34.6%] for the third child to 407 of 844 [48.2%] for the first child). Nearly all women (range, 352 of 389 [90.5%] for the second child to 797 of 844 [94.4%] for the first child) reported breastfeeding or breast milk pumping after returning to work, with many reporting insufficient time to do so (range, 33 of 78 [42.3%] for the third child to 453 of 844 [53.7%] for the first child) because of unpredictable schedules, inconvenience to patients, need to be present for training or for trainees, and inconvenience of breast pumping facilities. Approximately a quarter of participants reported experiencing discrimination related to breastfeeding or breast milk pumping (218 of 844 [25.8%] for the first child; 57 of 389 [14.7%] for the second child; and 7 of 78 [9.0%] for the third child) or inappropriate comments (257 of 844 [30.5%] for the first child; 73 of 389 [18.8%] for the second child; and 17 of 78 [21.8%] for the third child). The most frequently reported positive experience was emotional support (504 of 844 [59.7%] for the first child; 231 of 389 [59.4%] for the second child; and 51 of 78 [65.4%] for the third child), with colleagues being the leading source of all positive experiences, followed by supervisors, hospital staff, and other residents. For their first child, 152 respondents (18.0%) reported experiencing discrimination when returning to work, most often from colleagues, supervisors, a chief resident, or other residents. For subsequent children, fewer reported experiencing discrimination (52 of 389 [13.4%] for the second child; and 9 of 78 [11.5%] for the third child), but when it occurred, it was primarily from colleagues. For their first child, more than one-third of respondents (298 of 844 [35.3%]) experienced difficulty obtaining childcare, with a substantial percentage (214 of 844 [25.4%]) requiring additional support (ie, childcare support beyond a traditional workday).

**Table 3.** Experiences Returning to Work by Child

Experience	No. (%) of Respondents		
	Child 1 (n = 844)	Child 2 (n = 389)	Child 3 (n = 78)
Negative experiences upon return to work			
Verbal admonishment for increased workload on covering physicians	112 (13.3)	42 (18.8)	10 (12.8)
Discussion regarding anticipated delay in partnership status	17 (2.0)	13 (3.3)	3 (3.8)
Anger directed toward you in response to rescheduled appointments	55 (6.5)	21 (5.4)	7 (9.0)
Increased difficulty acquiring operating room times	11 (1.3)	9 (2.3)	0
Inability to acquire adequate facilities for pumping	272 (32.2)	80 (20.6)	12 (15.4)
Inadequate time for pumping	407 (48.2)	149 (38.3)	27 (34.6)
Inadequate frequency for pumping	372 (44.1)	127 (32.6)	24 (30.8)
Longer workdays	113 (13.4)	45 (11.6)	12 (15.4)
Derogatory words	60 (7.1)	27 (6.9)	3 (3.8)
Other	30 (3.6)	22 (5.7)	2 (2.6)
None	287 (34.0)	178 (45.8)	35 (44.9)
Experienced discrimination based on haven taken maternity/family leave^a^	152 (18.0)	52 (13.4)	9 (1.5)
Source of discrimination			
Family member	3 (2.0)	0	0
Colleague	56 (36.8)	25 (48.1)	5 (55.6)
Hospital/office staff	27 (17.8)	2 (3.8)	0 (0)
Administration	33 (21.7)	12 (23.1)	3 (33.3)
Chair	16 (10.5)	6 (11.5)	2 (22.2)
Mentor	9 (5.9)	2 (3.8)	1 (11.1)
Supervisor	47 (30.9)	11 (21.1)	4 (44.4)
Trainee	4 (2.6)	0	0
Patient	8 (5.3)	3 (5.8)	0
Chief resident	19 (12.5)	3 (5.8)	1 (11.1)
Attending	39 (25.7)	6 (11.5)	0
Nurses	9 (5.9)	3 (5.8)	0
Other residents	44 (28.9)	3 (5.8)	1 (11.1)
Other	2 (1.3)	1 (1.9)	0
Positive experiences return to work			
Emotional support	504 (59.7)	231 (59.4)	51 (65.4)
Mental support	171 (20.3)	84 (21.6)	24 (30.8)
Physical support	72 (8.5)	39 (10.0)	14 (17.9)
For pumping			
Adequate facilities	330 (39.1)	187 (48.1)	43 (55.1)
Adequate time	233 (27.6)	131 (33.7)	33 (42.3)
Adequate frequency	192 (22.7)	110 (28.3)	23 (29.5)
Flexible schedule	187 (22.2)	101 (26.0)	23 (29.5)
Other	14 (1.7)	7 (1.8)	2 (2.6)
None	163 (19.3)	69 (17.7)	8 (10.3)
Childcare			
Difficulty acquiring childcare			
Yes	298 (35.3)	91 (23.4)	12 (15.4)
No	490 (58.1)	275 (70.7)	61 (78.2)
NA	55 (6.5)	23 (5.9)	5 (6.4)
Type of childcare			
On-site daycare	54 (6.4)	24 (6.2)	5 (6.4)
Off-site daycare	302 (35.8)	145 (37.3)	28 (35.9)
Hired help (ie, nanny, babysitter)	316 (37.4)	166 (42.7)	34 (43.6)
Family member	254 (30.1)	93 (23.9)	22 (28.2)
Other	13 (1.5)	6 (1.5)	2 (2.6)
Required additional support (beyond standard childcare)	214 (27.2)	103 (28.1)	25 (34.2)
Breastfeeding/pumping			
Breastfed/pumped breast milk at work	797 (94.5)	352 (90.5)	73 (93.6)
Had appropriate facilities for breastfeeding/pumping	512 (60.7)	244 (69.3)	52 (71.2)
Insufficient time available for breastfeeding/pumping	453 (53.7)	168 (47.7)	33 (45.2)
Aware of federal laws pertaining to breastfeeding/pumping requirements for employees	566 (67.1)	258 (66.3)	55 (70.5)
Experienced discrimination due to breastfeeding/pumping	218 (32.2)	57 (16.2)	7 (9.6)
Experienced inappropriate comments or made to feel uncomfortable regarding breastfeeding/pumping	257 (27.4)	73 (20.7)	17 (23.3)

Abbreviation: NA, not applicable.

^a^ Percentages calculated using the total number of respondents who were actually asked the question.

## Discussion

Our study captured one of the largest and most heterogeneous samples of physician mothers to our knowledge, consisting of both attending physicians and residents from multiple specialties. The majority of respondents were attending physicians, and nearly all respondents were within their first 10 years of practice. Whereas previous literature has focused solely on individual specialties or residents, our study was across disciplines and levels of training and included experiences for each child conceived or adopted after medical school.

Participants in our study cited the need for more leave, particularly, as the most important support for physician mothers. Only a fraction of respondents felt that their leave time was sufficient, and the majority desired more leave time, ideally 4 to 6 months. More than one-third were less than satisfied with their maternity leave experience, and more than two-thirds were dissatisfied with the paternity leave available to their partner. Notably, a physician mother may have access to leave, but she may not take leave, with the most commonly cited reasons as pressure to return to clinical responsibilities, threat of prolonged training, and financial factors.

Regardless of which child they were reporting about, only approximately half of participants in our study reported having paid maternity/family leave offered by their employer through their benefits package. Similar to other studies, the primary source for paid maternity leave was accrued sick time followed by accrued paid time off.^5^ Several first-time mothers did not feel they had adequate information about the leave, consistent with other studies noting confusion regarding leave policies.^15,19^ Family leave policies should be clear and readily available, especially to first-time parents who may not be familiar with the federal rights to which they are entitled. Nearly half of respondents in our study had no paid leave. Less than half of respondents reported that their spouse or partner was offered paternity leave or domestic partner leave, and even when offered, very few spouses or domestic partners took that leave.

Return to work following maternity leave can be a difficult transition period owing to a variety of factors. Work-life integration is especially challenging in the early years after childbirth because of childcare availability and having to make up clinical time missed during leave. More than one-third of first-time mothers in our study reported difficulty acquiring childcare, and approximately one-fourth needed additional childcare support beyond standard childcare. Furthermore, more than one-third of the women in our study reported having to make up time taken for leave (eg, extra calls, extra clinic time) during the postpartum period, which can be a very emotionally and physically vulnerable time. Disproportionate responsibilities for childcare at home and punitive repercussions at work can lead to burnout, career dissatisfaction, and work-life imbalance, necessitating change in the ways we support physician mothers transitioning back into the workplace following maternity leave.^8,13,20^

Conscious and unconscious discrimination in the workplace presents another barrier to success and well-being for physician mothers.^13^ Our study supports this claim in multiple ways. First, several women in our study reported delaying their family planning, mostly due to financial constraints or threat of prolonged training, and some regretted this delay because they felt that it contributed to difficulties with conception. Women face a great deal of work-related conflict during graduate medical education, and residency programs may need to provide more effective support and accommodations if they want to continue to recruit and retain talented residents.^8,21,22,23^ Second, many physician mothers reported experiencing discrimination for having taken maternity/family leave, feeling pressured by their employer to return to work while on maternity leave, and receiving verbal admonishment for the higher workload for covering physicians. The source of this discrimination was predominantly colleagues, supervisors, other residents, and attending physicians. These figures are similar to sources of discrimination identified in one survey of orthopedic residents^24^ but differ from other studies that cite nurses as a primary source of discrimination.^13^ Third, nearly all respondents breastfed or pumped breast milk at work; however, lack of access to lactation facilities and especially lack of adequate time for pumping breast milk were cited as the most common negative experiences on return to work, consistent with previous literature.^25,26,27^ Lactation support is not only a vital asset to physician mothers, but also a legal requirement that is often not accessible to women in medicine because of unpredictable schedules and inconvenience for patients.

Despite documented problems for many physician mothers in the workplace,^23,24^ there is also evidence that not all women have negative experiences. For example, just over half of the women in our study reported having uninterrupted time to focus on their new child during leave and experiencing emotional support on return to work from colleagues, supervisors, staff, and fellow residents.

### Limitations

We were unable to track how many potentially eligible individuals had access to the survey link; thus, we could not calculate a response rate. In addition, as with any surveys administered electronically, several participants exited the survey prematurely. There were many incomplete surveys received, and we elected to not include any participant’s data that could not be reasonably assumed to be complete. The length of the survey may have contributed to the number of incomplete surveys and overall number of participants; however, the total sample size was relatively large given the depth and breadth of data collected. The retrospective nature of this survey study may have resulted in recall bias. Also, we recruited through social media and electronic listservs, which may have produced a sampling bias if women who are part of these social media and professional groups differ systematically from those who are not.

## Conclusions

To our knowledge, the present study represents one of the most heterogeneous samples of physician mothers assessed through a rigorously developed survey aiming to capture experiences surrounding maternity leave and return to work. Our study showed that women physicians from a variety of specialties at all levels of training across the United States needed and wanted more support for maternity leave and return to work. Although policy changes are being made at the state level, support at the institutional level, such as paid leave, adequate breast milk pumping time without penalty, on-site childcare, and schedule flexibility, would likely have the greatest direct effect on women in medicine, as suggested by our results. Our survey yielded extensive data on both the negative and positive experiences of physician mothers, identifying areas in which change is needed and strategies for successfully supporting physician parents. We created a rich data set of quantitative and qualitative information about the experiences of women physicians across multiple specialties to address a wide variety of questions to inform policy and to advocate for changes necessary for physician mothers to thrive in their careers.

Physician burnout and dissatisfaction are becoming increasingly problematic owing to demanding schedules and inadequate support for achieving work-life integration.^1,2,6,8^ At the same time, the face of medicine is changing, with women representing an increasing proportion of US practicing physicians.^3^ Because having children is central to the health, well-being, and work-life balance for a large number of women in medicine, we need to identify best policies and practices to support physician mothers while maintaining quality clinical care. Physicians taking extended leave present unique challenges for both institutions and individuals, making the answers for how best to support women in medicine far from simple.^4^ These challenges require creative solutions, the seeds of which can be found in the lived experiences of women in medicine. Our study captures these lived experiences, creating an opportunity to identify both problems and effective strategies associated with maternity/family leave and return to work for physician parents.
